# Uso de romiplostim en trombocitopenia inmunitaria: experiencia en Cuenca (Ecuador)

**DOI:** 10.7705/biomedica.7059

**Published:** 2024-05-31

**Authors:** Héctor Chiang-Wong, Patricio González-Saldaña

**Affiliations:** 1 Unidad de Hematología, Hospital José Carrasco Arteaga, Cuenca, Ecuador Hospital José Carrasco Arteaga Hospital José Carrasco Arteaga Cuenca Cuenca; 2 Unidad de Patología Clínica, Hospital José Carrasco Arteaga, Cuenca, Ecuador Hospital José Carrasco Arteaga Hospital José Carrasco Arteaga Cuenca Cuenca

**Keywords:** trombocitopenia, trombopoyetina, Ecuador, thrombocytopenia, thrombopoietin, Ecuador

## Abstract

**Introducción.:**

El consenso internacional y la guía del 2019 de la *American Society of Hematology*, establecieron a los análogos de la trombopoyetina como medicamentos de segunda línea para tratar la trombocitopenia inmunitaria primaria. En Ecuador, se comercializan dos trombomiméticos: romiplostim y eltrombopag.

**Objetivos.:**

Describir el uso de romiplostim en pacientes con trombocitopenia inmunitaria, en un hospital de tercer nivel en Cuenca (Ecuador).

**Materiales y métodos.:**

Se adelantó un estudio descriptivo y retrospectivo en pacientes con trombocitopenia inmunitaria y tratamiento con romiplostim. Se evaluaron las siguientes variables: edad, sexo, tratamientos previos a romiplostim, dosis, frecuencia, complicaciones, cambio de análogo de trombopoyetina y discontinuación de la terapia.

**Resultados.:**

Veintiún pacientes con trombocitopenia inmunitaria fueron tratados con romiplostim, con una mediana de 49 años. Todos recibieron corticoides como tratamiento de primera línea. Tres precisaron de intervalos más prolongados que el semanal, con dosis semanales menores de las recomendadas (< 1 μg/kg). Por falta de eficacia, en seis pacientes se reemplazó la terapia con eltrombopag por romiplostim. Tres pacientes padecieron complicaciones trombóticas: dos, trombosis venosa portal, y uno, tromboembolia_pulmonar. En cinco, se discontinuó el tratamiento con romiplostim, sin necesidad de reanudarlo.

**Conclusiones.:**

Romiplostim constituye un tratamiento de segunda línea para la trombocitopenia inmunitaria primaria. A pesar del reducido tamaño de la muestra, se observó que la administración temprana del medicamento puede minimizar toxicidades y riesgos infecciosos.

## Abstract (en)

**Introduction.:**

The international consensus and the American Society of Hematology guidelines from 2019 established thrombopoietin analogues as the second-line therapy for primary immune thrombocytopenia cases.

**Objectives.:**

To describe romiplostim usefulness in patients with immune thrombocytopenia in a third-level hospital in Cuenca, Ecuador.

**Materials and methods.:**

We conducted a descriptive and retrospective study in patients with immune thrombocytopenia treated with romiplostim. We evaluated the following variables: age, gender, previous therapies to romiplostim, dose, frequency, complications, change of thrombopoietin analogue, and treatment discontinuation.

**Results.:**

We included 21 patients with immune thrombocytopenia treated with romiplostim, with a median age of 49 years. All patients received corticosteroids as first-line treatment. Three patients required longer administration intervals (over a week), with weekly doses lower than those recommended (< 1 μg/kg). Due to lack of efficacy, six patients replaced elthrombopag with romiplostim. Of the total, three suffered thrombotic complications: two had portal venous thrombosis, and one had pulmonary thromboembolism; five of the patients discontinued romiplostim scheme without resuming it.

**Conclusions.:**

Romiplostim constitutes a convenient second-line therapy in immune thrombocytopenia. Despite the small sample size, romiplostim early use can minimize toxicities and infectious risks.

La trombocitopenia inmunitaria primaria una es enfermedad adquirida en la cual solo del 10 al 30 % de los pacientes muestra una mejoría clínica sostenida después del retiro de los corticoides como tratamiento de primera línea ^1^. En la actualidad, no existe consenso sobre cómo secuenciar o escalonar los tratamientos de segunda línea, entre los que se encuentran los análogos de la trombopoyetina o trombomiméticos, el rituximab o la esplenectomía. La elección se basa en la duración de la enfermedad, la presencia de comorbilidades, la edad del paciente, los costos y la disponibilidad del tratamiento.

La *Food and Drug Administration* (FDA) y la *European Medicines Agency* (EMA), han aprobado tres trombomiméticos para el manejo de la trombocitopenia inmunitaria primaria: romiplostim, eltrombopag y avatrombopag. Estos alcanzan tasas de mejoría clínica global del 75 al 80 % y son sostenidas en el 60 % de la población si su administración continúa por seis meses ^1^. En Ecuador, se comercializan dos tipos de trombomiméticos: romiplostim y eltrombopag.

A continuación, se presenta una serie de casos con 21 pacientes que requirieron romiplostim en un hospital de tercer nivel en Ecuador, con el objetivo de establecer su perfil de aplicación clínica y alternativa terapéutica.

## Materiales y métodos

Se desarrolló un estudio descriptivo y retrospectivo en pacientes mayores de quince años con trombocitopenia inmunitaria tratados con romiplostim entre agosto del 2015 y febrero del 2023. Se incluyeron pacientes con lupus eritematoso sistémico.

Los datos se recopilaron a partir de las historias clínicas digitalizadas de un hospital de tercer nivel en Cuenca (Ecuador). Se evaluaron las siguientes variables: edad, sexo, fases de trombocitopenia inmunitaria primaria, tratamientos previos al romiplostim, dosis, frecuencia, complicaciones, cambio de análogo de la trombopoyetina y discontinuación del tratamiento.

Con base en las guías de la *American Society of Hematology* del 2019, se establecieron las fases de la trombocitopenia inmunitaria primaria como de reciente diagnóstico (evolución inferior a tres meses), persistente (de tres meses a un año) y crónica (de un año o más).

Se consideró como ‘mejoría sin tratamiento con romiplostim’ un recuento plaquetario de 50.000 células/μl o más durante mínimo seis meses (veinticuatro semanas consecutivas) en ausencia de romiplostim u otro tratamiento concomitante o de rescate para trombocitopenia inmunitaria primaria.

El trabajo fue avalado por el Comité de Bioética del Hospital José Carrasco Arteaga de Cuenca (Ecuador).

### Análisis estadístico

Los datos de las variables descriptivas se recabaron con el programa Excel (Microsoft, Redmond, WA) y se analizaron con el *software* SPSS™, versión 25.0 para Windows (Chicago, IL). Se emplearon: la prueba U de Mann-Whitney para el análisis bivariado de contraste de dos medias independientes; el método de Kaplan-Meier, para los análisis de supervivencia, y el test de log *rank* (Mantel-Cox), para evaluar diferencias entre grupos. Se consideró como estadísticamente significativo un valor inferior a 0,05.

## Resultados

Se revirtieron los porcentajes a números absolutos dado que el tamaño de la muestra es pequeño (menor de 40) y a que pueden prestarse para interpretaciones erróneas.

Veintiún pacientes con trombocitopenia inmunitaria fueron tratados con romiplostim, con una mediana de edad de 49 años (IC_95%_: 38,70-58,04), distribución multimodal y 12 de sexo femenino. El paciente de menor edad tenía 17 años y, el de mayor edad, 90 años.

Previa administración de romiplostim, todos los pacientes recibieron corticoides; 11 se sometieron a esplenectomía y a 14 se les prescribió rituximab. Cinco pacientes tenían lupus eritematoso sistémico.

Cuatro de los pacientes sufrían de trombocitopenia inmunitaria primaria de reciente diagnóstico, en seis, era persistente y, en once, de evolución crónica. La mediana de tiempo fue de 93 meses para la administración de romiplostim (Q_1_ = 51,5 y Q_3_ = 106,5) desde el diagnóstico de trombocitopenia inmunitaria primaria.

A dieciocho pacientes se les administró su dosis con frecuencia semanal; sin embargo, tres requirieron intervalos más prolongados (más de una semana), con dosis menores de las referenciadas en la ficha técnica (< 1 μg/kg a la semana). Dos recibieron la dosis máxima permitida (10 μg/kg a la semana).

Tres pacientes presentaron complicaciones trombóticas, como trombosis venosa portal (n = 2) y tromboembolia_pulmonar (n = 1).

En seis pacientes se cumplieron los criterios de ‘mejoría sin tratamiento con romiplostim’, con una mediana de seguimiento de siete meses (Q_1_ = 3,5 y Q_3_ = 27,1). Para este análisis, se excluyeron cinco pacientes por cambio a eltrombopag (n = 3), pérdida de seguimiento por la pandemia sanitaria (n = 1) y por falta de mejoría clínica (n = 1). En cinco se pudo discontinuar el romiplostim sin reanudarlo.

**Figura 1:**
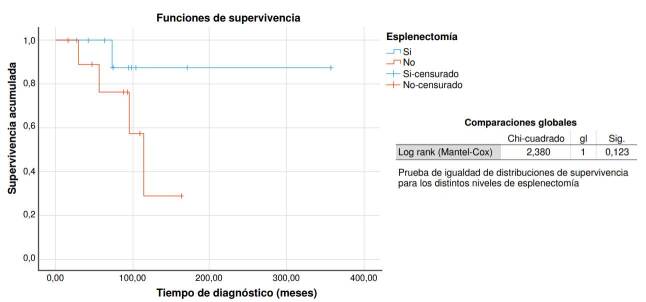
Comparación de curvas de supervivencia entre pacientes esplenectomizados y no esplenectomizados

No se encontró evidencia estadísticamente significativa al comparar las variables evaluadas de los que continuaron con romiplostim y los que cumplieron los criterios de mejoría clínica (cuadro 1); a diferencia de los que recibieron rituximab y se sometieron a esplenectomía (figura 1 y 2).

**Cuadro 1:** Resultados de las pruebas U de Mann-Whitney

Variable	p
Edad	1,000
Sexo	0,885
Tiempo de diagnóstico (meses)	0,563
Tiempo de tratamiento (meses)	0,186
Esplenectomía	0,105
Rituximab	0,724
Eltrombopag	0,635
Otros medicamentos	0,351
Frecuencia de romiplostin	0,683
Dosis (> 50.000 plaquetas/µl)	0,048
Lupus eritematoso sistémico	0,342
Trombosis	0,683
Trombocitosis	0,952
Clasificación de la trombocitopenia inmunitaria primaria	0,085

**Figura 2:**
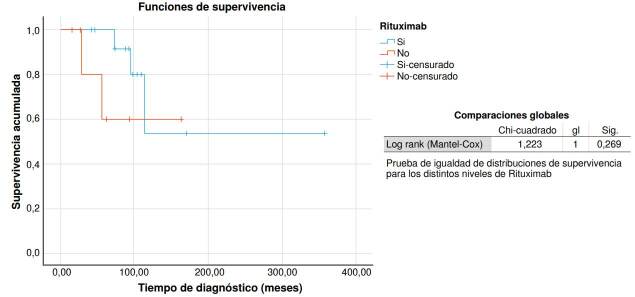
Comparación de curvas de supervivencia entre pacientes que recibieron rituximab y los que no lo recibieron

## Discusión

Pocos hospitales públicos en Ecuador acceden a los análogos de la trombopoyetina, por lo cual el rituximab y la esplenectomía continúan siendo las alternativas de manejo. La terapia “puente” con trombomiméticos previa a la esplenectomía, constituye una estrategia válida, si no se ha cumplido el año de diagnóstico.

En un análisis retrospectivo de 194 pacientes con trombocitopenia inmunitaria primaria y sometidos a esplenectomía por vía laparoscópica, Zychowicz *et al*. demostraron que el grupo con recuentos por debajo de 50.000 plaquetas/μl (9,09 %) no se asoció con tasas altas de complicaciones, en comparación con el grupo con recuentos por encima de 50.000 plaquetas/ μl (11,51 %) ^2^. Entre las complicaciones principales, se destacaron: neumonía (1,81 %), hematoma (1,81 %), absceso (1,81 %), sangrado (1,81 %) y pancreatitis aguda (1,81 %). En el presente estudio, los pacientes desestimados para esplenectomía tenían una edad superior a los 65 años, antecedente de lupus eritematoso sistémico o negaron el consentimiento.

Deshayes *et al*. llevaron a cabo un estudio prospectivo multicéntrico de 248 pacientes con trombocitopenia inmunitaria primaria, tratados con rituximab, en el cual valoraron su eficacia y seguridad a largo plazo. Con un seguimiento de 60 meses, 73 pacientes (29,4 %) presentaron una mejoría clínica sostenida, mientras 24 tuvieron mejoría inicial con posterior recaída. Estos últimos fueron tratados nuevamente con rituximab y presentaron una mejoría del 92 % con una duración mayor del 54 % ^3^.

No hay estudios controlados aleatorizados que contrasten romiplostim con eltrombopag, pero sus tasas de mejoría clínica son similares. La administración de romiplostim es subcutánea y semanal, con una dosis inicial de 1 a 3 μg/kg a la semana, hasta una máxima de 10 μg/kg a la semana, con el objetivo de lograr recuentos plaquetarios estables mayores de 50.000 células/μl. Por otro lado, la administración de eltrombopag es oral (preferiblemente con el estómago vacío), diaria, con una dosis inicial de 50 mg/día (25 mg en casos de pacientes asiáticos o con hepatopatías), hasta una máxima de 75 mg/día.

La farmacocinética del romiplostim limita el empleo de intervalos de aplicación mayores a la semana, excepto si están en proceso de suspensión o con dosis mínimas ^1^. En el presente estudio, tres pacientes desarrollaron trombocitosis, a pesar de recibir dosis menores de las recomendadas (< 1 μg/kg a la semana).

Romiplostim refleja mayor fluctuación plaquetaria que eltrombopag, quizá por los períodos extendidos de uso o la absorción subcutánea errática. En los pacientes evaluados, se formularon puntos de corte plaquetarios mayores de los sugeridos en la ficha técnica, con el propósito de minimizar las fluctuaciones en la reducción de las dosis (200.000 plaquetas/μl) y la suspensión transitoria del medicamento (400.000 plaquetas/μl). Asimismo, al requerirse una suspensión de romiplostim de más de una semana, se aconsejó administrar, de manera gradual, del 10 al 25 % más de la dosis respecto a la última aplicada, para impedir una trombocitopenia de rebote.

El intercambio entre los análogos de la trombopoyetina fue efectivo para producir mejoría clínica solo en 50-80 %, lo que contrarresta la hipótesis de resistencia cruzada ^2^^-^^6^. En el presente trabajo, cuatro pacientes se cambiaron de romiplostim a eltrombopag por la dificultad de asistir al hospital para su administración, debido a su horario laboral o la lejanía del lugar de residencia; sin embargo, uno de ellos fue tratado nuevamente con romiplostim por falta de mejoría clínica. De ocho pacientes que recibieron inicialmente eltrombopag, seis se cambiaron a romiplostim por falta de eficacia del primero. Un paciente no mejoró con ninguno de los dos análogos de la trombopoyetina.

En un estudio retrospectivo de 303 pacientes, Ito *et al*. demostraron que la incidencia acumulativa de trombosis -arterial y venosa- 5 y 10 años después del uso de análogos de la trombopoyetina fue del 5,3 y el 10 %, respectivamente ^7^^-^^9^.

Kuter *et al*. identificaron un incremento en la tasa de eventos tromboembólicos en los pacientes con trombocitopenia inmunitaria primaria crónica que recibieron romiplostim (IC_95%_: 6,1 por 100 pacientes al año), respecto a aquellos con trombocitopenia inmunitaria primaria persistente o de reciente diagnóstico (IC_95%_: 4,4 por 100 pacientes al año) ^8^.

Tres de los pacientes del presente estudio presentaron eventos trombóticos: uno presentó tromboembolia pulmonar (sin factores de riesgo) y dos padecieron trombosis venosa portal (un paciente con lupus eritematoso sistémico y el otro sin factores de riesgo).

En un estudio prospectivo de fase IV, Janssens *et al*. evaluaron la incidencia de la reticulina y el colágeno en las biopsias de médula ósea de 131 pacientes con trombocitopenia inmunitaria primaria que recibieron romiplostim después del primer, del segundo o del tercer año de administración. El 6,9 % tuvo un incremento mayor, igual a dos grados en la escala de Bauermeister. Sin embargo, en la práctica clínica habitual, en el hospital se carece de criterios de vigilancia para la fibrosis medular ^10^.

Zaha *et al*. recopilaron trabajos clínicos de discontinuación de análogos de la trombopoyetina con sus porcentajes de mejoría clínica sin tratamiento: Leven *et al*. (33 %), Mahevas *et al*. (15 %), Cervinek *et al*. (24 %), González-López *et al*. (100 %), Newland *et al*. (75 %) y Marshall *et al*. (28 %) ^11^. En el presente estudio, en cinco de los 21 pacientes se pudo discontinuar la terapia con romiplostim sin necesidad de reanudarlo.

Con la carencia de factores predictivos, se recomienda evitar el retiro del romiplostim si el paciente ^11^^-^^14^ presenta recuentos plaquetarios fluctuantes (< 30.000-50.000 células/μl) con dosis intermedias o altas, si el tiempo de administración es de seis meses o más o, si recibe simultáneamente otros medicamentos para la trombocitopenia (como corticoides), si recibe antiagregantes o anticoagulantes con plaquetas por debajo de las 100.000 células/μl, o si tiene comorbilidades que repercutan en la calidad de vida.

En la actualidad, catorce pacientes con trombocitopenia inmunitaria se benefician con análogos de la trombopoyetina en el hospital José Carrasco Arteaga: diez con romiplostim y cuatro con eltrombopag.

En el presente trabajo, se analizaron las características demográficas y las tasas de discontinuación de análogos de la trombopoyetina, semejantes a las descritas en la literatura, con predominio del sexo femenino y una mediana de edad entre la cuarta y quinta década de la vida.

Entre las limitaciones del estudio, se encuentra su carácter retrospectivo, el tamaño reducido de la muestra y su ejecución en un solo centro hospitalario.

Por la similitud de los sistemas sanitarios, podría crearse un registro de pacientes a nivel latinoamericano, esencial para establecer el mejor perfil clínico de aplicación de estos fármacos y su impacto económico.

En conclusión, el romiplostim es conveniente y seguro como medicamento de segunda línea para tratar la trombocitopenia inmunitaria primaria. A pesar del tamaño reducido de la muestra, se observó que la administración temprana del medicamento puede minimizar toxicidades y riesgos infecciosos.

## References

[1] Álvarez-Román MT, Berrueco-Moreno R, Canaro-Hirnyk M, Entrena-Ureña L, Fernández-Fuertes F, González-López T (2020). Directrices de diagnóstico, tratamiento y seguimiento de la PTI. Recomendaciones del Grupo de Trabajo de la SEHH y GEPTI.

[2] Zychowicz A Radkowiak, D Lasek A Malczak P, Witowsky J Major P (2018). Laparoscopic splenectomy for thrombocytopenia immune in patients with a very low platelet count. Videosurgery Miniinv.

[3] Deshayes S, Khellaf M, Zarour A, Layese R, Fain O, Terriou L (2019). Long-term safety and efficacy of rituximab in 248 adults with immune thrombocytopenia: Results at 5 years from the French prospective registry ITP-ritux. Am J Hematol.

[4] González-Porras J, Godeau B, Carpenedo M (2019). Switching thrombopoietin receptor agonist treatments in patients with primary immune thrombocytopenia. Ther Adv Hematol.

[5] Álvarez-Román MT, Fernández-Bello I, Arias-Salgado EG, Rivas-Pollmar MI, Jiménez-Yuste V, Martín-Salces M (2014). Effects of thrombopoietin receptor agonists on procoagulant state in patients with immune thrombocytopenia. Thromb Haemost.

[6] Provan D, Arnold D, Bussel J, Chong B, Cooper N, Gernsheimer T (2019). Updated international consensus report on the investigation and management of primary immune thrombocytopenia. Blood Adv.

[7] Ghanima W, Cooper N, Rodeghiero F, Godeau B, Bussel J. (2018). Thrombopoietin receptor agonists: Ten years later. Haematologica.

[8] Kuter DJ, Newland A, Chong B, Rodeghiero F, Romero M, Pabinger I (2019). Romiplostim in adult patients with newly diagnosed or persistent immune thrombocytopenia (ITP) for up to 1 year and in those with chronic ITP for more than 1 year: A subgroup analysis of integrated data from completed Romiplostim studies. Br J Haematol.

[9] Ito S, Fujiwara S, Ikeda T, Toda Y, Mashima K, Umino K (2020). Evaluation of thrombotic events in patients with immune thrombocytopenia. Ann Hematol.

[10] Janssens A, Rodeghiero F, Anderson D, Chong B, Boda Z, Pabinger I (2016). Changes in bone marrow morphology in adults receiving Romiplostim for the treatment of thrombocytopenia associated with primary immune thrombocytopenia. Ann Hematol.

[11] Zaja F, Carpenedo M, Barate C, Borchiellini A, Chiurazzi F, Finazzi G (2020). Tapering and discontinuation of thrombopoietin receptor agonists in immune thrombocytopenia: Real- world recommendations. Blood Rev.

[12] Cooper N, Hill Q, Grainger J, Westwod J, Bradbury C, Provan D (2021). Tapering and discontinuation of thrombopoietin receptor agonist therapy in patients with immune thrombocytopenia: Results from a modified Delphi Panel. Acta Haematol.

[13] Neunert C, Terrel D, Arnold D, Buchanan G, Cines D, Cooper N (2019). American Society of Hematology 2019 guidelines for immune thrombocytopenia. Blood Adv.

[14] Lino M, Sakamoto Y, Sato T. (2020). Treatmentfree remission after thrombopoietin receptor agonist discontinuation in patients with newly diagnosed immune thrombocytopenia: An observational retrospective analysis in realworld clinical practice. Int J Hematol.

